# A comprehensive evaluation of association between homocysteine levels and single nucleotide polymorphisms with hypertension risk: A protocol of systematic review and network meta-analysis

**DOI:** 10.1097/MD.0000000000020791

**Published:** 2020-06-26

**Authors:** Yixuan Kong, Jinghui Zheng, Xiangmei Xu, Xuan Chen, Jie Wang, Liying Lu, Zhuomiao Ye

**Affiliations:** a Ruikang Affiliated Hospital of Guangxi University of Chinese Medicine; b Department of Geriatrics, Ruikang Affiliated Hospital of Guangxi University of Chinese Medicine, Nanning, Guangxi, China.

**Keywords:** homocysteine, hypertension, network meta-analysis, single nucleotide polymorphisms, susceptibility

## Abstract

**Background:**

According to the relevant reports that single nucleotide polymorphisms (SNPs) may contribute to change of homocysteine (HCY) levels and increase the risk of hypertension (HTN). During the inconsistent results, this meta-analysis purpose is systematically review and synthesized relevant data on HCY levels and SNPs in HTN.

**Methods:**

The systematic search database, from the following database to find out the association studies of SNPs and HTN publications up until March 2020 from the databases of PubMed, Embase, Web of Science, Cochrane Library, China National Knowledge Infrastructure (CNKI), the Chinese Science and Technology Periodical Database (VIP) and Wan fang databases, and Chinese Biomedical Literature Database (CBM). Network meta-analysis and Thakkinstian's algorithm were used to select the most appropriate genetic model, along with false positive report probability (FPRP) for noteworthy associations. All statistical analyses were calculated with STATA software (version 14.0; StataCorp, College Station, TX).

**Results:**

This meta-analysis will provide high-quality evidence to the effects of SNP on HTN and levels of HCY, and find between SNPs and HTN susceptibility on in all the genetic models, and choose the best one.

**Conclusions:**

This meta-analysis will research which SNP is the most correlated with HTN risk.

**Registration:**

INPLASY202050002

## Abstract 2

Supplemental Digital Content is available in the text

## 1 Introduction

Hypertension (HTN) is defined as systemic blood pressure higher than 140 mm Hg, diastolic blood pressure higher than 90 mm Hg, or both.^[1]^ According to related research states that HTN is affecting more than 1 billion people worldwide, and it is estimated that more than 29.2% of adults will be considered HTN in 2025.^[2]^ It is accompanied by cardiovascular risks and high prevalence, leading to HTN has become one of the important causes of mortality from cardiovascular diseases^[2,3]^; and the pivotal source of “loss of health” worldwide.^[4]^

A large amount of environmental and clinical risk factors are associated with HTN, including sodium intake, alcohol intake, lack of exercise, poor diet, obesity, insulin resistant diabetes, and hyperlipidemia.^[5]^ Although these factors explain a considerable part of HTN susceptibility; but, genes, environmental factors, and their interactions have significant impact on the pathological process of HTN.^[6]^ It is evaluated that up to 60% of the variation in HTN risk is caused by an individual's genetic makeup.^[7]^

Single nucleotide polymorphism (SNPs) is a common genetic variation in a population, with the discovery frequency over 1%.^[8]^ Previously, a lot of research have shown that homocysteine (HCY) is an independent risk factor for cardiovascular and cerebrovascular diseases.^[9]^ From the perspective of Physiology, HCY may increase blood pressure by increasing arteriolar contraction, renal sodium reabsorption, renal dysfunction, activity of sympathetic nervous system, activity of rennin–angiotensin–aldosterone system, and arterial stiffness.^[10]^ The Third National Health and Nutrition Examination Survey (NHANES III) reported that the prevalence rate of HTN in people with the highest level of HCY is 2 to 3 times higher than whose with the lowest HCY level.^[11]^ HCY is a sulfhydryl-containing amino acid intermediate, which is the result of demethylation of methionine, an essential amino acid derived from protein and endogenous protein metabolism in diet.^[12]^ The four major enzymes involved in HCY metabolism are methylenetetrahydro-folate reductase (MTHFR), methionine synthase (MS), methionine synthase reductase (MTRR), methione synthase reduce (MSR), and cystathionine β-synthase (CBS).

Therefore, gene polymorphism may influence the level of HCY and is closely associated with the occurrence of HTN. Previously, many epidemiological studies have explored the relationship between gene polymorphism and HTN, but the results were inconsistent. This meta-analysis will provide a high-quality evidence to the effects of SNP on HTN and levels of HCY, and find between SNPs and HTN susceptibility on in all the genetic models, and choose the best one.

## 2 Materials and methods

This study was conducted in accordance with the PRISMA (Preferred Reporting Items for Systematic Reviews and Meta-Analyses) guidelines and the protocol was registered in the INPLASY (INPLASY202050002).

### 2.1 Identification of eligible studies

We carried out a systematic search that compared frequency differences in SNPs between HTN patients and healthy controls were search in PubMed, Embase, Web of Science, Cochrane Library, China National Knowledge Infrastructure (CNKI), the Chinese Science and Technology Periodical Database (VIP) and Wanfang databases, and Chinese Biomedical Literature Database (CBM) as of March 2020. The search strategy was based on the following search terms: “single nucleotide polymorphism,” “SNP,” “homocysteine,” “hypertension,” and “network meta-analysis.” Details regarding the search terms are available in the Supplementary Materials.

### 2.2 Inclusion criteria

Studies in this meta-analysis must meet the following inclusion criteria:

1. Types of patients: people with HTN;
2. Intervention: SNPs;
3. Types of comparisons: Healthy population;
4. Study designs: case–control study;
5. No restrictions were placed on age, gender, country, or type of HTN
6. When there were multiple same research publications from the same research team, only the largest study was included.

### 2.3 Exclusion criteria

Excluded studies had to meet the following criteria

1. duplication report, conference report, thesis, review paper, or animal study, poster
2. Studies without detailed genotypic distribution data
3. Studies in which SNPs demonstrated a departure from Hardy–Weinberg equilibrium (HWE) in controls

### 2.4 Data extraction and analysis

**Figure 1:**
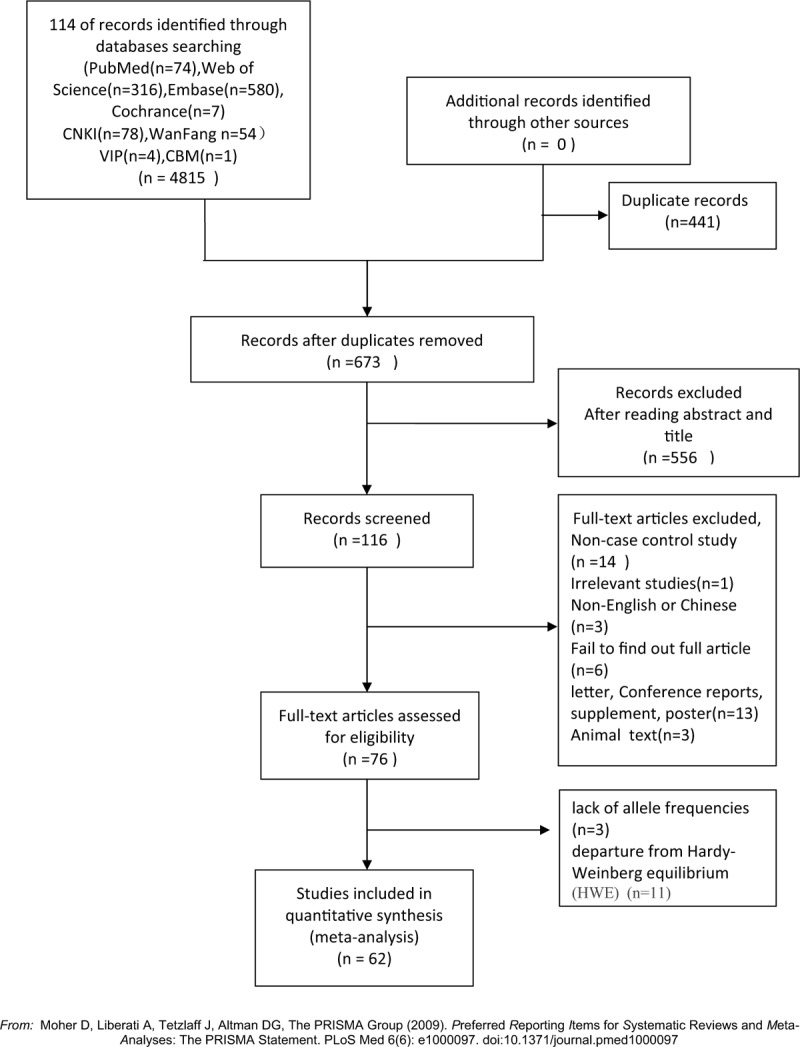
PRISMA flow diagram of literature search and selection.

The eligible data were extracted from the included studies by two investigators (Wang Jie and Xu Xiangmei) independently, resolved the disagreement by negotiate or consultation with a third investigator (Zheng Jinghui) (Fig. 1 is the PRISMA flow diagram illustrating the procedure of study selection). The following data from studies were selected: First author's name, publication year, methods of gene detection, country, region, race, number of cases and controls, genotype, and gene frequency distribution of case and controls and Hardy–Weinberg equilibrium (HWE) of controls.

Hardy–Weinberg equilibrium was evaluated for each study by Chi-square test in control groups, and *P* < .05 was considered a significant departure from HWE. Only selected studies that conform to HWE. We analyzed six models: allele contrast model, homozygous model, heterozygous model, dominant model, recessive mode, and additive model. Calculated fixed or random effects pooled odds ratio (OR) with 95% confidence intervals (CIs) for pairwise meta-analysis, choose statistically significant models, Stata software 15.1 is used for data processing. Heterogeneity was quantified with the *I*^2^ statistic and *P* value; a *I*^2^ statistic <50% and a *P* > .1 indicated low heterogeneity between studies, in which case the fixed-effect model (based on Mantel–Haenszel method) was employed; otherwise, the random-effects model (based on DerSimonian–Laird method) was adopted. Statistical heterogeneity among the studies was checked by chi-square-based *Q*-test. When indicating substantial heterogeneity, we find out the source through subgroup analysis.

Second, a random-effects network meta-analysis was conducted using ADDIS 1.16.7 within a Bayesian framework. Four Markov chain carries out initial value setting, the variance scaling factor is 2.5, the iteration step length is refined by 10, the tuning iteration number is adjusted by 20,000, and the simulation iteration number is 50,000. When the potential scale reduction factor PSRF tends to 1, convergence was satisfied. When significant deviations were detected, we use an inconsistency model; otherwise, the consistency model was used. This Bayesian approach was used to rank the probability of each genetic model for risk assessment and generate corresponding ranking probability maps. Thakkinstian's criteria were also used to select the most appropriate genetic model. Meta-DiSc software was used for diagnostic meta-analysis to determine the sensitivity and specificity of SNPs in predicting HTN risks. Evaluate the correlation between the combined sensitivity and specificity using the summarized receiver operating characteristic (SROC) curve and its area under curve (AUC); positive likelihood ratio (+LR), negative likelihood ratio (−LR), and diagnostic dominance ratio (DOR) were calculated accordingly.

### 2.5 Qualitative evaluation

The qualities of the included studies were accessed based on the STREGA statement.^[13]^ Two investigators respectively according to the methodological quality assessment, the third investigator resolved the disagreement.

### 2.6 Subgroup analysis

If heterogeneity is found to be caused by certain factors, we will conduct subgroup analysis; according to age, race, gender, and type of HTN.

### 2.7 Sensitivity analysis

Sensitivity analysis will be conducted to check the each study robustness and reliability of pooled outcome result.

### 2.8 Reporting bias

Using the Begg's and Egger's tests to detect publication bias.

## 3 Discussion

A lot of studies proposed that the levels of HCY in hypertensive were higher than in subjects without HTN. However, numerous epidemiological studies have explored the relationship of the polymorphism with HTN, but the results were inconsistent. In this network meta-analysis, we explored the relationship between gene polymorphism and HTN through four key metabolic enzymes in the metabolic pathways of HCY: MTHFR, MSR, CBS, MTRR, Six kinds of genetic models are established. Through comprehensive analysis of different genetic models, the prediction accuracy is improved and the deviation of single model prediction is reduced. For the pairwise meta-analysis, we have screened out models that are meaningful to this study. To identify the most appropriate model for HTN risk association, Thakkinstian's algorithm were used; along with false positive report probability (FPRP) for noteworthy associations. This meta-analysis will provide a high-quality evidence to the effects of SNP on HTN and levels of HCY, and find between SNPs and HTN susceptibility. Further investigations with larger sample sizes and more ethnic groups are carried out to confirm these results, to provide an evidence base for public health management of HTN and guiding clinical practice.

## Author contributions

Conceived and designed this study: Kong Yixuan. Performed database retrieval: Wang Jie and Xu Xiangmei. Data processing: Chen Xuan, Lu Liying, and Ye Zhuomiao. Read, provided feedback and approved the final manuscript: Zheng Jinghui. All authors approved the final version of the manuscript.

## Supplementary Material

Supplemental Digital Content

## References

[1] Chobanian AV Bakris GL Black HR The 7th report of the joint national committee on prevention, detection, evaluation, and treatment of high blood pressure: the JNC 7 report. J Am Med Assoc 2003;289:2560–72. 10.1001/jama.289.19.2560 12748199

[2] Patricia MK Megan W Kristi R Global burden of hypertension: analysis of worldwide data. Lancet (London, England) 2005;365:217–23. 10.1016/S0140-6736(05)17741-1 15652604

[3] Lewington S Clarke R Qizilbash N Age-specific relevance of usual blood pressure to vascular mortality: a meta-analysis of individual data for one million adults in 61 prospective studies. Lancet 2002;360:1903–13. 12493255 10.1016/s0140-6736(02)11911-8

[4] Agustin G-V Fara S Monzon CM Thick ascending limb sodium transport in the pathogenesis of hypertension. Physiol Rev 2019;99:235–309. 30354966 10.1152/physrev.00055.2017 PMC6335098

[5] Fowdar JY Lason MV Szvetko AL Investigation of homocysteine-pathway-related variants in essential hypertension. Int J hypertens 2012;2012: doi: 10.1155/2012/190923. 10.1155/2012/190923 PMC3485977 23133742

[6] Fu L Li YN Luo D Evidence on the causal link between homocysteine and hypertension from a meta-analysis of 40 173 individuals implementing Mendelian randomization. J Clin Hypertens (Greenwich) 2019;21:1879–94. 31769183 10.1111/jch.13737 PMC8030561

[7] Singh M Mensah GA Bakris G Pathogenesis and clinical physiology of hypertension. Cardiol Clin 2010;28:545–59. 20937440 10.1016/j.ccl.2010.07.001

[8] Schröder NW Schumann RR Single nucleotide polymorphisms of toll-like receptors and susceptibility to infectious disease. Lancet Infect Dis 2005;5:156–64. 15766650 10.1016/S1473-3099(05)01308-3

[9] Petramala L Acca M Francucci CM Hyperhomocysteinemia: a biochemical link between bone and cardiovascular system diseases? J Endocrinol Invest 2009;32: Suppl 4: 10–4. 19724160

[10] Stehouwer CD van Guldener C Does homocysteine cause hypertension? Clin Chem Lab Med 2003;41:1408–11. 14656018 10.1515/CCLM.2003.216

[11] Lim U Cassano PA Homocysteine and blood pressure in the 3rd National Health and Nutrition Examination Survey, 1988–1994. Am J Epidemiol 2002;156:1105–13. 12480655 10.1093/aje/kwf157

[12] Chai AU Abrams J Homocysteine: a new cardiac risk factor? Clin Cardiol 2001;24:80–4. 11195611 10.1002/clc.4960240113 PMC6655194

[13] Little J Higgins JP Ioannidis JP Strengthening the reporting of genetic association studies (STREGA): an extension of the STROBE statement. Hum Genet 2009;125:131–51. 19184668 10.1007/s00439-008-0592-7

