# Supplementary material for: A comprehensive evaluation of association between homocysteine levels and single nucleotide polymorphisms with hypertension risk: A protocol of systematic review and network meta-analysis
Source: Medicine (Baltimore). 2020 Jun 26;99(26):e20791. doi: 10.1097/MD.0000000000020791 (PMC7328956; doi:10.1097/MD.0000000000020791)
Supplement: Supplemental Digital Content [file medi-99-e20791-s001.docx]

((((("Polymorphism, Genetic"[Mesh]) OR "Polymorphism, Single Nucleotide"[Mesh]) OR "Genotype"[Mesh]) OR "Alleles"[Mesh])) AND (("Hypertension"[Mesh]) AND ("Homocysteine"[Mesh]))
